# Association analysis of ADPRT1, AKR1B1, RAGE, GFPT2 and PAI-1 gene polymorphisms with chronic renal insufficiency among Asian Indians with type-2 diabetes

**DOI:** 10.1186/1471-2350-11-52

**Published:** 2010-03-31

**Authors:** Pushplata Prasad, Arun K Tiwari, KM Prasanna Kumar, AC Ammini, Arvind Gupta, Rajeev Gupta, BK Thelma

**Affiliations:** 1 Department of Genetics, University of Delhi South Campus, New Delhi, India; 2 Department of Endocrinology and Metabolism, MS Ramiah Medical College, Bangalore, India; 3 Department of Endocrinology, All India Institute of Medical Sciences, New Delhi, India; 4 Jaipur Diabetes and Research Centre, Jaipur, India; 5 Monilek Hospital and Research Centre, Jaipur, India

## Abstract

**Background:**

To determine association of nine single nucleotide polymorphisms (SNPs) in ADP ribosyltransferase-1 (ADPRT1), aldo-keto reductase family 1 member B1 (AKR1B1), receptor for advanced glycation end-products (RAGE), glutamine:fructose-6-phosphate amidotransferase-2 (GFPT2), and plasminogen activator inhibitor-1 (PAI-1) genes with chronic renal insufficiency (CRI) among Asian Indians with type 2 diabetes; and to identify epistatic interactionss between genes from the present study and those from renin-angiotensin-aldosterone system (RAAS), and chemokine-cytokine, dopaminergic and oxidative stress pathways (previously investigated using the same sample set).

**Methods:**

Type 2 diabetes subjects with CRI (serum creatinine ≥3.0 mg/dl) constituted the cases (n = 196), and ethnicity and age matched individuals with diabetes for a duration of ≥ 10 years, normal renal functions and normoalbuminuria recruited as controls (n = 225). Allelic and genotypic constitution of 10 polymorphisms (SNPs) from five genes namely- *ADPRT1*, *AKR1B1, RAGE, GFPT2 *and *PAI-1 *with diabetic CRI was investigated. The genetic associations were evaluated by computation of odds ratio and 95% confidence interval. Multiple logistic regression analysis was carried out to correlate various clinical parameters with genotypes, and to study epistatic interactions between SNPs in different genes.

**Results:**

Single nucleotide polymorphisms -429 T>C in *RAGE *and rs7725 C>T SNP in 3' UTR in *GFPT2 *gene showed a trend towards association with diabetic CRI. Investigation using miRBase statistical tool revealed that rs7725 in *GFPT2 *was a perfect target for predicted miRNA (hsa miR-378) suggesting the presence of the variant 'T' allele may result in an upregulation of GFPT2 contributing to diabetic renal complication. Epistatic interaction between SNPs in transforming growth factor *TGF-β1 *(investigated using the same sample set and reported elsewhere) and *GFPT2 *genotype was observed.

**Conclusions:**

Association of SNPs in *RAGE *and *GFPT2 *suggest that the genes involved in modulation of oxidative pathway could be major contributor to diabetic chronic renal insufficiency. In addition, GFPT2 mediated overproduction of TGF-β1 leading to endothelial expansion and thereby CRI seems likely, suggested by our observation of a significant interaction between GFPT2 with TGF-β1 genes. Further, identification of predicted miRNA targets spanning the associated SNP in *GFPT2 *implicates the rs7725 SNP in transcriptional regulation of the gene, and suggests *GFPT2 *could be a relevant target for pharmacological intervention. Larger replication studies are needed to confirm these observations.

## Background

Diabetic chronic renal insufficiency (CRI) is the leading cause of death due to end stage renal disease world over. Glycemic control and blood pressure along with duration of diabetes are major risk factors for development of this micro-vascular complication. Familial clustering and the observation that diabetic nephropathy afflicts only 20-30% of all diabetic patients, indicate a genetic component underlying disease development. Further, prevalence of diabetic CRI is also known to vary between populations, with non-Caucasian diabetic patients having higher risk than Caucasians [1]. Polymorphisms in genes from several biochemical pathways such as aldose reductase-polyol, di-acyl glycerol-protein kinase C, non-enzymatic glycation and glycoxidation, hexosamine pathway, and renin-angiotensin-aldosterone system (RAAS) have been implicated in the development of diabetic chronic renal insufficiency [2]. Many of these pathway genes have been previously tested for association with CRI in the Indian population [3-6]. On the other hand, in experimental models [7,8] and clinical trials [9] the inhibitors of these individual pathways failed to block various downstream events leading to disease development, suggesting that all these hyperglycemia mediated pathways could possibly be linked to a common upstream event. An increase in oxidative stress through overproduction of superoxide radical by the mitochondrial electron transport chain was proposed to be that common event and the initiating factor in diabetic kidney disease [2]. Excess production of superoxide activates all the above mentioned pathways through deoxyribonucleic acid (DNA) damage mediated activation of poly adenosine diphosphate (ADP) ribose polymerase (PARP) causing inhibition of glyceraldehyde-3-phosphate dehydrogenase (GAPDH), a key glycolytic enzyme. It has been proposed that the decreased GAPDH activity upregulates the polyol pathway, increases intracellular advanced glycation end-product (AGE) formation, activates protein kinase C (PKC), and hexosamine pathway flux. In addition, hexosamine pathway mediates activation of the plasminogen activator inhibitor-1 (PAI-1) promoter in vascular smooth muscle cells which is a major factor causing endothelial dysfunction and initiates diabetes related complications [2].

Variants in ADP-ribosyltransferase-1 (ADPRT1), aldo-keto reductase family 1 member B1 (AKR1B1), receptor for advanced glycation end products (RAGE), plasminogen activator inhibitor-1 (PAI- 1) and glutamine:fructose-6-phosphate amidotransferase 2 (GFPT2) genes have been investigated for their association with different disease conditions. Val762Ala and Phe54Leu of the *ADPRT1 *gene encoding PARP have been tested for association with chronic glomerulonephritis in Russian patients [10]. *AKR1B1 *variants have been shown to be associated with diabetic neuropathy [11,12], nephropathy [13,14] and retinopathy [1,15]. Polymorphisms in RAGE gene have been implicated for type-1 diabetes, type-2 diabetes and their complications in Caucasian populations [16-18]. PAI-1 gene polymorphisms are widely implicated in development of hypertension and obesity. However, a few reports also indicate their influence in development of diabetes and its complications [19-27]. Genetic variants in GFPT2 variants are associated with type 2 diabetes and diabetic nephropathy [28].

We investigated the association of nine functionally significant polymorphisms in this set of five oxidation pathways modulation genes (*ADPRT1, AKR1B1, RAGE, GFPT2 AND PAI-1*) with CRI among Asian Indians with type 2 diabetes. Further, it is well known that a complex disease is a manifestation of interaction between candidate genes from different biological pathways. Numerous cellular pathways (including hexosamine, cytokine-chemokine, advanced glycation end product, aldose reductase, polyol, and renin-angiotensin-aldosterone system) are activated by increased glucose levels and their interaction with each other is believed to influence development and progression of diabetic CRI [2]. Therefore, in the present report, which is the last of the series from our laboratory, we attempted to identify putative pathological epistatic interactions between genes from multiple pathways using a combined data set from this study together with data from all our previous studies carried out using the same sample set on RAAS [3], chemokine [4], dopaminergic [5], and oxidative stress [6] genes.

In addition, based on available biological explanations, independent assessments for epistatic interactions between glutamine: fructose-6-phosphate amidotransferase (*GFPT2*) and transforming growth factor -*β*1 (*TGF-β1*); and angiotensin converting enzyme (ACE) and plasminogen activator inhibitor-1 (*PAI-1*) were also carried out. Under long standing hyperglycemia, the hexosamine pathway is induced by shunting of excess intracellular glucose where Fructose-6 phosphate is metabolized into *N*-acetyl-glucosamine and thereafter metabolized to glycolipids, proteoglycans and glycoproteins. The rate limiting enzyme in hexosamine biosynthesis is GFPT. Increased concentration of GFPT in mesangial cells enhances formation of proteoglycans and glycoproteins which upregulates transcription of TGF-β1, a known pro-sclerotic agent, and an important causative factor for development of diabetic nephropathy [29].

For interaction between SNP pair in ACE and PAI-1 genes, the renin-angiotensin-aldosterone system (RAAS) is speculated to interact with the plasminogen activator inhibitor (PAI-1) system for regulation of fibrinolysis and intercellular matrix modeling. ACE is the rate limiting enzyme of the RAAS. A beneficial effect of ACE inhibitor on diabetic kidney disease has been observed in clinical trials, which underscores the importance of ACE in diabetic CRI. The 300 bp insertion/deletion (I/D) polymorphism in intron 16 of ACE has been widely implicated in diabetic CRI across several populations. Further, the ACE *DD *genotype was found to be associated with increased PAI-1 activity [30], and ACE inhibition was found to attenuate the peak circulating level of PAI-1 [31]. Interaction between ACE with PAI-1 gene polymorphisms was found to decrease PAI-1 mRNA expression in irradiated kidney with a role in modification of intercellular matrix modeling and renal damage [32]. With this background, in this study, we investigated epistatic interactions between TGF-β1 promoter (-800 G>A) SNP and rs7725, C>T in GFPT2; and ACE I/D polymorphism and homozygous Del (4G/4G) marker of PAI-1 gene pairs.

## Methods

### Subjects

Complete clinical and demographic characteristics of the study population have been reported elsewhere (3, Table 1). Briefly, in this retrospective case-control study consecutive consenting subjects suffering from type-2 diabetes with CRI defined as serum creatinine ≥3 mg/dl (n = 196), and diabetics without any evidence of diabetic kidney disease (diabetes of more than 10 years, normal renal function, no significant proteinuria; n = 225) were recruited from the outpatient departments of the participating medical institutions situated across India. The study was approved by respective institutional ethics committee and an informed consent was obtained from all the participants. 10 ml venous blood was collected from each individual included in the study for biochemical and genetic analysis. Biochemical analyses to determine fasting glucose, glycated hemoglobin, serum creatinine, triglycerides, total cholesterol, and albumin were carried out at the respective centres using automated analyzers. Using serum creatinine as a surrogate marker we calculated glomerular filtration rate (GFR) by using the online modified diet in renal disease (MDRD) calculator http://www.nephron.com/cgi-bin/MDRD_GFR.cgi[33]. For CRI, the inclusion criteria were subjects with type 2 diabetes for ≥ 2 years, plus two or more of the following: serum creatinine ≥ 150 umol/l, urinary albumin excretion rate (AER) > 200 mg/l, and presence of retinopathy. Normoalbuminuric (AER<20 mg/l) type-2 diabetes subjects of ≥ 10 years duration of diabetes (mean duration 17.07 ± 6.69 years) were recruited as control diabetes subjects. An aliquot of blood from the four centers was transported to the genetics laboratory for DNA isolation and amplification.

**Table 1:** Clinical characteristics of the study population

Characteristics	DM^a^ (N = 225)	CRI^b^ (N = 196)	P
Gender (M/F)	76/149	65/131	0.89^d^
Age (years)	60.6 ± 11.5	57 ± 12.8	0.10^c^
Duration of Diabetes (years)	17.07 ± 6.69	10.4 ± 7.7	<0.05^c^
Hb A_1c _(%)	7.3 ± 1.0	7.5 ± 1.1	0.949^c^
Systolic pressure (mm Hg) *	140 (106-190)	150 (110-210)	0.002^e^
Diastolic pressure (mm Hg) *	84 (80-104)	90 (70-110)	0.025^e^
Serum creatinine (μmol/l) *	84 (35-108)	177 (124-1112)	<<0.05^e^
^f^UAER (mg/l) *	10 (1-16)	864 (320-1584)	<<0.05^e^
^g^GFR (mls/min/1.73 m^2^)	81.44 ± 21.85	27.83 ± 24	<<0.05^c^
Serum triglyceride (mmol/l)	1.7 ± 0.67	1.8 ± 1.4	0.307^c^
Serum cholesterol (mmol/l)	4.8 ± 0.97	4.9 ± 0.92	0.852^c^
Retinopathy (%)	24	88	
Non proliferative (%)	15	35	
Proliferative (%)	8.6	53	<<0.05^d^
Cardiovascular events (%)	3	8.3	

Data presented as mean ± SD (* median and range). ^a^type 2 diabetes subjects without nephropathy (DM); ^b^with diabetic renal insufficiency (CRI); ^c^Student's t test; ^d^Pearson's χ^2 ^test; ^e^Mann-Whitney U test, ^f^Urinary albumin excretion rate, ^g^Glomerular filtration rate.

### Genetic analysis

A total of 10 polymorphisms from five candidate genes namely- *ADPRT1, AKR1B1, RAGE, GFPT2 *and *PAI-1 *were genotyped in DM (controls) and CRI (cases) subjects (Table 2). SNPs were selected based on their minor allele frequency and functional relevance: SNPs leading to missense mutations, promoter polymorphisms, and those present in the regulatory regions (5' UTR and 3' UTR) were chosen in the same order of priority. Genotyping was done using either polymerase chain reaction (PCR) restriction fragment length polymorphisms (RFLP) or PCR-length polymorphism (LP) approach. Digested PCR products were resolved on 2-3% agarose gel stained with ethidium bromide. The PCR primers, PCR and RFLP conditions and genotypic profiles obtained for each of the polymorphisms are presented in Table 2.

**Table 2:** Polymorphisms in *ADPRT1, AKR1B1, RAGE, GFPT2 and PAI-1 *genes, their location, primer sequences, PCR conditions and restriction enzyme with product sizes.

Gene, OMIM No., nucleotide change, position and rs ID of the polymorphism	Primer sequence	Product size (bp)	Annealing temp./Restriction enzyme/allele sizes
**ADPRT1 **(*173870; 1q42.12) C>G (Phe54Leu; **rs3738708**)	F: 5'- TCGAGGTCAAGGTCAAGGTC -3' R: 5'- CTGTCACTCCTCCAGCTTCC -3'	249	60°C/BsaX I/ C = 106,143 bp G = 249
**ADPRT1 **(*173870; 1q42.12) T>C (Val762Ala; rs1136410)	F: 5'- CCC AAA TGT CAG CAT GTA CG -3' R: 5'- AGG CCT GAC CCT GTT ACC TT -3'	406	57°C/Ssi I/ T = 406 bp C = 268 bp, 138 bp
**ADPRT1 **(*173870; 1q42.12) A>G (Arg940Lys; rs3219145)	F: 5'- AGA TGG TCT GGG TTT TGT GG-3' R: 5'- TGA ACC TCC AAT CAT GGT CA -3'	399	57°C/Eco9I1/ A = 399 bp G = 243 bp,156 bp
**AKR1B1 **(*103880; 7q35) 5' UTR G>C (rs5053)	F: 5'-ACT AGG ACC AGG CGG AAG AA -3' R: 5'- CCG TTG TTG AGC AGG AGA C-3'	234	58°C/Msp I/ G = 154 bp, 80 bp C = 234 bp
**AKR1B1 **(*103880; 7q35) 5' UTR, G>A (rs759853)	F: 5'-ACT AGG ACC AGG CGG AAG AA -3' R: 5'- CCG TTG TTG AGC AGG AGA C-3'	234	58°C/BfaI/ G = 234 bp A = 204 bp, 20 bp
**RAGE **(*600214; 6p21.3) -374T>A (rs1800624)	F: 5'- AAA ACA TGA GAA ACC CCA GA-3' R: 5'-CCC CGA TCC TAT TTA TTC CA -3'	222	57°C/Alu I/ T = 222 bp A = 176 bp, 46 bp
**RAGE **(*600214; 6p21.3) -429T>C (rs1800625)	F: 5'- AAA ACA TGA GAA ACC CCA GA-3' R: 5'-CCC CGA TCC TAT TTA TTC CA -3'	222	57°C/Tas I/ T = 121 bp, 101 bp A = 222 bp
**RAGE **(*600214; 6p21.3) Promoter, 63 bp Ins/Del polymorphism	F: 5'- AAA ACA TGA GAA ACC CCA GA-3' R: 5'-CCC CGA TCC TAT TTA TTC CA -3'	222	57°C/ Ins = 222 bp Del = 159 bp
**GFPT2 **(*603865; 5q34-q35 3'UTR, C>T (rs7725)	F5'-GGCTTTCTGTAGCCGTGGT-3' R5'-TACTATGGGCAAGGAGCAG-3'	361 bp	58°C/Tsp509I/ C = 361 bp T = 218,143 bp
**PAI-1 **(*173360; 7q21.3) Promoter, PAI-1 4G/5G	F5'-CACAGAGAGAGTCTGGACACGT-3' R5'- CCCAACAGAGGACTCTTGGTC-3'	101 bp	62°C/Bsl I 4G = 97 bp,4 bp 5G = 74 bp, 23 bp,4 bp

### Statistical Analysis

Comparison of all clinical variables between DM and CRI subjects were carried out by χ^2 ^test for nominal variables or t-test for continuous variables. Hardy-Weinberg equilibrium was tested for each of the SNPs based on the genotyping of 220 normal healthy individuals (average age 35.11 ± 8.98 years). These were recruited on a random basis from different locations including public meeting places, offices, colleges, markets and hospitals and represent population based controls. Allelic and genotypic associations of polymorphisms were evaluated by Pearson's χ^2 ^test/Fisher's exact test followed by odds ratio (OR) and 95% confidence intervals (CI) computation. P values < 0.05 were considered significant. Power of the sample size for each of the SNPs at 5% significance level was calculated using PAWE software version 1.2 [34,35]. Linear and multiple logistic regression (Backward) analyses were carried out to correlate various clinical parameters with genotypes, and gene-gene interactions between SNPs of different pathways. Additive inheritance model in multiple regression analysis was employed to test interaction between SNP pairs from different pathways.

The miRBase::Sequence tool was used to identify miRNA Target in 3'UTR of gene. The .miRBase::Sequence Database is a searchable database of published miRNA sequences and annotation [miRBase::Sequences http://microrna.sanger.ac.uk/sequences]. Each entry in the miRBase Sequence database represents a predicted hairpin portion of a miRNA transcript (termed mir in the database), with information on the location and sequence of the mature miRNA sequence (termed miR).

## Results

All the investigations in this study were carried out on the single sample set (cases 196, controls 225) reported earlier [3]. Clinical characteristics of the two groups are shown in Table 1.

### Genetic analyses

Polymorphism C>G (rs3738708) in *ADPRT1*, G>C (rs5053) in *AKR1B1 *and -374T>A in *RAGE *were found to be monomorphic in this population and not analyzed further. Remaining seven markers were in Hardy Weinberg Equilibrium in the study population. Results of allelic and genotypic association are presented in Table 3. Bonferroni correction value for the univariate association analysis in this study was (β) = 0.05/7 = 0.007. None of the association observed in the study withstood the Bonferroni correction.

**Table 3:** Allele and genotype frequencies of SNPs in *ADPRT1, AKR1B1, RAGE, GFPT2, PAI *and their association status with diabetic CRI, and the power of sample size to detect association at 5% significance level.

SNPs	Allele frequency		Genotype frequency		Association		Power (G %)
	DM	CRI	DM	CRI	Allele	Genotype	
	(n = 225) (n = 196)		(n = 225)	(n = 196)	(df = 1)	(df = 2)	
**ADPRT1** **T>C**	T = 0.91 C = 0.09	T = 0.925 C = 0.075	TT = 0.87 TC = 0.12 CC = 0.01	TT = 0.83 TC = 0.16 CC = 0.01	χ^2 ^= 0.21; P = 0.64, P* = 0.13	χ^2 ^= 1.61; P = 0.45; P* = 0.11	12
**ADPRT1** **A>G**	A = 0.91 G = 0.09	A = 0.92 G = 0.08	AA = 0.83 AG = 0.17 GG = 0.00	AA = 0.84 AG = 0.14 GG = 0.02	χ^2 ^= 0.07; P = 0.79; P* = 0.13	χ^2 ^= 3.3; P = 0.19; P* = 0.14	8
**AKR1B1** **G>C**	G = 0.93 C = 0.07	G = 0.93 C = 0.07	GG = 0.86 GC = 0.14 CC = 0.00	GG = 0.89 GC = 0.11 CC = 0.00	χ^2 ^= 0.88; P = 0.35; P* = 0.15	χ^2 ^= 0.94; P = 0.33 (df = 1); P* = 0.14	0
**RAGE** **-429T>C**	T = 0.87 C = 0.13	T = 0.88 C = 0.12	TT = 0.77 TC = 0.22 CC = 0.01	TT = 0.81 TC = 0.14 CC = 0.05	χ^2 ^= 0.04; P = 0.84; P* = 0.11	**χ^2 ^= 8.68;** **P = 0.013;** P* = 0.015 **OR = 9.03, 95% CI = (1.09<OR<74.26)**	7
**RAGE** **63 bp ins/del**	Ins = 0.96 Del = 0.04	Ins = 0.97 Del = 0.03	Ins/Ins = 0.95 Ins/Del = 0.05 Del/Del = 0.00	Ins/Ins = 0.91 Ins/Del = 0.09 Del/Del = 0.00	χ^2 ^= 0.26; P = 0.60; P* = 0.15	P* = 0.06	12
**GFPT2** **C>T**	C = 0.78 T = 0.22	C = 0.72 T = 0.28	CC = 0.50 CT = 0.45 TT = 0.05	CC = 0.60 CT = 0.37 TT = 0.03	**χ^2 ^= 3.92;** **P = 0.047;** P* = 0.03 **OR = 1.38, 95% CI = (1.02<OR<1.98)**	χ^2 ^= 4.5; P = 0.11; P* = 0.04	51
**PAI-1 4G/5G (del/ins)**	4G = 0.52 5G = 0.48	4G = 0.49 5G = 0.51	4G4G = 0.23 4G5G = 0.52 5G5G = 0.25	4G4G = 0.29 4G5G = 0.46 5G5G = 0.25	χ^2 ^= 0.84; P = 0.36; P* = 0.06	χ^2 ^= 2.5; P* = 0.29	14

P:P value for Pearson's chi-square test

P*: Fisher's exact P value.

#### ADPRT1

No allelic or genotypic association of T>C (Val>Ala; rs1136410) and A>G (Arg>Lys, rs3214915) SNPs with diabetic CRI was observed (Table 3).

#### AKR1B1

No allelic or genotypic association of the G>A (rs759853) polymorphism with CRI in our sample set was seen (Table 3).

#### RAGE

No allelic association of the -429T>C, 63 bp Ins/Del polymorphisms with diabetic CRI was observed (Table 3). However, genotype CC of -429T>C SNP exhibited statistical association on odds ratio calculation (OR: 9.03, 95%CI: 1.09-74.26).

#### GFPT2

A marginal allelic association of 3' UTR polymorphism (rs7725, C>T) with CRI was observed in this study (Table 3). Allele T seems to confer susceptibility to CRI (OR: 1.38, 95%CI: 1.02-1.98, P = 0.047). On further investigation using the miRBase tool it was observed that 3' UTR polymorphism (rs7725) was a perfect target for predicted miRNA (hsa miR-378).

#### PAI-1

No allelic or genotypic association of PAI-4G/5G (deletion/insertion polymorphism) with diabetic CRI was observed (Table 3).

Multivariate logistic regression analysis (backward method) was carried out using disease status as a dependent variable, and age, gender, BMI, duration of diabetes, GFR and genotypes of all seven polymorphisms as independent variables. 3' UTR polymorphism (rs7725, C>T) (OR = 1.44, 95%CI 2.02-1.02, P = 0.036) in *GFPT2 *was found to be associated with diabetic CRI.

Assessment of epistatic interaction between candidate genes from different biological pathways, including RAAS, chemokine, dopaminergic, and oxidative stress pathways was performed using backward MLR (SPSS11.0). A significant interaction between GG genotype (of -800 G>A SNP in TGFβ1 gene) and CC genotype (of rs7725, C>T in GFPT2 gene) was observed in our sample set. A combination of GG and CC genotype seems to confer protection (OR = 0.63, 95% CI 0.40-0.97, P = 0.035) against CRI. Gene-gene interactions analyzed using complete data set from this study and all our previous reports reiterated results of pair wise interaction between TGF β1 and GFPT2 genes.

GFR (glomerular filtration rate) is a linearly distributed clinical parameter crucial in determining the extent of kidney damage, and therefore may act as a shadow marker for kidney disease. Therefore, a linear regression analysis was carried out using GFR values as a dependent variable and various genetic markers and clinical parameters (age, gender, BMI, duration of diabetes) as independent variables. We did not observe association of any of the independent variables with GFR.

## Discussion

Association studies using polymorphisms in genes from classical pathways such as aldose reductase-polyol, di-acyl glycerol-protein kinase C, non-enzymatic glycation/glycoxidation, hexosamine pathway and renin-angiotensin-aldosterone system (RAAS) have identified certain major and minor susceptibility modifiers which contribute to development of diabetic chronic renal insufficiency [36-38]. Contemporarily it is believed that an upstream oxidative stress event may be the master switch regulating all the above mentioned hyperglycemia mediated pathways and thus mediate development and progression of diabetic kidney disease [2]. However, like in all other complex traits, the results have been inconsistent across studies. Therefore, considering the importance of ADPRT1 as marker of PARP, AKR1B1, RAGE, GFPT2 and PAI-1 genes in regulation of superoxide production, association analysis of functionally significant variants in these genes becomes imperative. Present analysis indicates that SNPs -429 T>C in RAGE and rs7725 C>T in 3' UTR in GFPT2 gene (Table 3) could be probable genetic contributor to CRI.

Micro- and macrovascular diabetic complications are influenced by formation of advanced glycation end products (AGEs) [39], which occur at an accelerated rate under long standing hyperglycemia. AGEs alter vascular functions by their formation on the extracellular matrix altering vascular structure and trapping circulatory proteins, leading to a narrowing of the lumen [39,40]. In addition, formation of AGEs on intracellular proteins and DNA leads to cellular changes. The principle means of derangement of AGEs is by specific AGE-binding receptors, which include the AGE-receptor complex, the macrophage scavenger receptors, and the receptor for AGE (RAGE). Latter is a member of immunoglobulin super-family located at chromosome 6p21.3 in the MHC locus [41] and is the most widely implicated by linkage/association studies. Major up-regulation of RAGE in vasculature has been observed in diabetic individuals and animal models in the presence of vascular disease. Animal models engineered to over-express RAGE develop diabetic nephropathy more rapidly than their normal counterparts [42].

The two functional promoter polymorphisms (-374 T>A and -429 T>C) in *RAGE *are known to alter transcription levels. -374T allele has been seen to confer protection against chronic heart disease, ischemic heart disease and atherosclerosis in diabetic patients [43]. This marker notably is monomorphic in our population. 10% of the samples were re-genotyped to rule out genotyping error. On the other hand CC genotype of -429 T>C SNP is predisposing (OR: 9.03, 95% CI: 1.09 - 74.26) to diabetic CRI. This observation derives support from an earlier report of an increased expression of RAGE gene in the presence of -429C, -374A and 63 bp D alleles [44]. A heterozygote excess of this marker in our control (DM) group may be masking the allelic association of this variant. Further, the very low frequency (0.01) of CC homozygotes in the control group may be suggestive of its potential nature to confer major susceptibility to severe kidney impairments. However, the power to detect association of this SNP is very low (G = 7%) and therefore should not be over-interpreted. To overcome the limitation posed by small sample size and to replicate our result we are currently genotyping all the SNPs analysed in the study on a larger sample set.

Increased flux of glucose through the hexosamine biosynthetic pathway has been implicated in diabetes and diabetic nephropathy. The rate-limiting enzyme is glutamine: fructose-6-phosphate amidotransferase (GFPT 2), which catalyzes the formation of glucosamine-6-phosphate from glucosamine and fructose-6-phosphate. Up-regulation of hexosamine pathway has been seen to influence TGF β1 production in the mesangial cells of rat kidney [45,46], which is a key molecule in the pathogenesis of diabetic nephropathy. A study involving African-American subjects reported a significant association of 'T' allele of the 3' UTR SNP with diabetic nephropathy [28]. With this knowledge, the trend of association of 'T' allele of rs7725 in 3' UTR in GFPT2 that we observed is exciting and warrants further investigation. 'T' allele of rs7725, C>T in GFPT2 has been shown to be approximately 2-fold over-expressed resulting in increased mRNA levels with resultant increased hexosamine flux [28]; which in turn has been speculated to increase extra-cellular matrix (ECM) synthesis and accumulation finally leading to loss of kidney function [47]. Such a transcriptional control exerted by this 3'UTR SNP may now be convincingly explained by the recent knowledge that the wild type allele of this SNP is a perfect target site for predicted miRNA (hsa miR - 378) using miRBase::Sequences tool http://microrna.sanger.ac.uk/sequences. The presence of the variant 'T' allele may result in up-regulation of GFPT2.

As for the contribution of TGF β1 gene, we have previously reported a significant association of a compound group of genotypes 'GA and AA' of SNP G>A -800 in TGF β1 gene which was found to be predisposing (P = 0.035; OR: 3.028; CI: 1.079-8.50) and GG was found to be protective against CRI in a subset of the patients with proliferative retinopathy [4]. TGF β1 has been known to influence almost every pathway implicated in development of diabetic CRI [4]. Considering the above discussed role of both GFPT 2 and TGF-β1 in pathogenesis of diabetic nephropathy, our findings of gene-gene interactions of TGF-β1 and GFPT2 genotypes may suggest that these two genes are major determinants of CRI in our population (Please see result section). Notwithstanding these interesting findings in this study which fulfils most of the criteria of a good genetic association analysis [3], the moderate sample size and absence of replication in an independent cohort may pose as study limitations.

## Conclusions

This study indicates a trend towards association of -429 T>C polymorphism in RAGE and rs7725 in the 3' UTR in GFPT2 genes with diabetic renal disease in Indian subjects. The study also shows a significant gene-gene interaction between *GFPT 2 *and *TGF-β1*. These observations are patho-physiologically important and warrant replication in a larger sample set.

## Competing interests

The authors declare that they have no competing interests.

## Authors' contributions

All authors have read and approved the final Ms. PP was involved in the study design, carried out molecular genetics and statistical analyses, compiled the data, wrote the Ms.; AKT was involved in molecular genetic analysis; KMPK, ACA, AG, AKS, and RG were the principal clinical investigators involved in study design, defining exclusion and inclusion criteria of study subjects and were mainly responsible for identification of study subjects from their respective clinical centres; BKT was the principal geneticist and coordinator of the project, involved in conceptualization of the project, study design, oversee complete genetic analyses in the laboratory, critical inputs and finalization of the manuscript.

## Pre-publication history

The pre-publication history for this paper can be accessed here:

http://www.biomedcentral.com/1471-2350/11/52/prepub
